# Biosignature reservoirs in the Rozel Point tar seeps at Great Salt Lake, Utah

**DOI:** 10.3389/fmicb.2026.1832944

**Published:** 2026-08-03

**Authors:** Cayla Martin, Mary Lilibeth Sanchez, June Baxter, David L. Parrott, Bonnie K. Baxter

**Affiliations:** 1 Great Salt Lake Institute, Westminster University, Salt Lake City, UT, United States; 2 Center for Fundamental and Applied Microbiomics and School of Life Sciences, Arizona State University, Tempe, AZ, United States

**Keywords:** asphalt, biosignature, extremophile, Great Salt Lake, methane, petroleum

## Abstract

On the north shore of Great Salt Lake, Utah, USA, naturally occurring tar seeps at Rozel Point provide an opportunity to study a petroleum-rich environment adjacent to a hypersaline desert ecosystem. Currently, this terminal lake is shrinking, and the Rozel tar spreads along the surface of the dry lakebed, forming seeps that are numerous and variable in size. These seeps result from high molecular weight hydrocarbons migrating up through cracks and fissures along faulted basalt and limestone. Our gas analysis in the seeps revealed emissions of methane and carbon dioxide, suggesting microbial activities such as petroleum degradation and methanogenesis. To probe the microbial diversity, we collected Rozel petroleum samples from three distinctive seeps and extracted DNA then probed the taxonomic diversity with specific primers (16S rRNA genes of bacteria and archaea; 18S rRNA genes of eukaryotes; Internal Transcribed Spacers 1 and 2 for fungi). Sequencing and a phylogenetic analysis demonstrated a robust microbial community in the tar including halophilic species originating from the nearby lake water and sediment as well as species that may degrade the petroleum. However, most of the bacterial signals were indicative of animal-related microbiota, likely introduced by the tar-entrapped fauna or from the surrounding ranchland. A large portion of the fungal biosignatures were related to plant-associated species from the local steppe. Taken together, the Rozel tar seeps capture a unique snapshot of the biology in the area, which may be a compelling rationale for studying tar seeps on Earth as biosignature traps. Also, we share astrobiology insights for considering potential life on other space bodies: this hydrocarbon reservoir may serve as an analog for studies of similar biosignature-collecting asphalt seeps.

## Graphical Abstract

**Figure:**
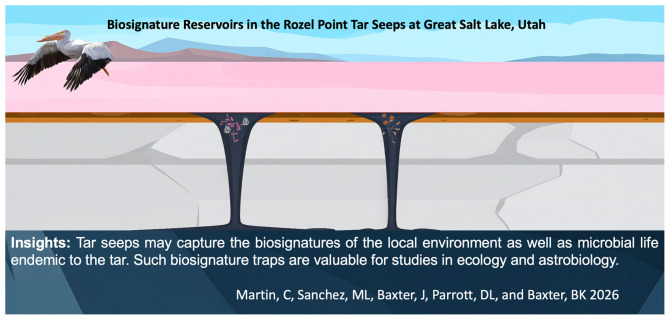

## 1 Introduction

**FIGURE 1:**
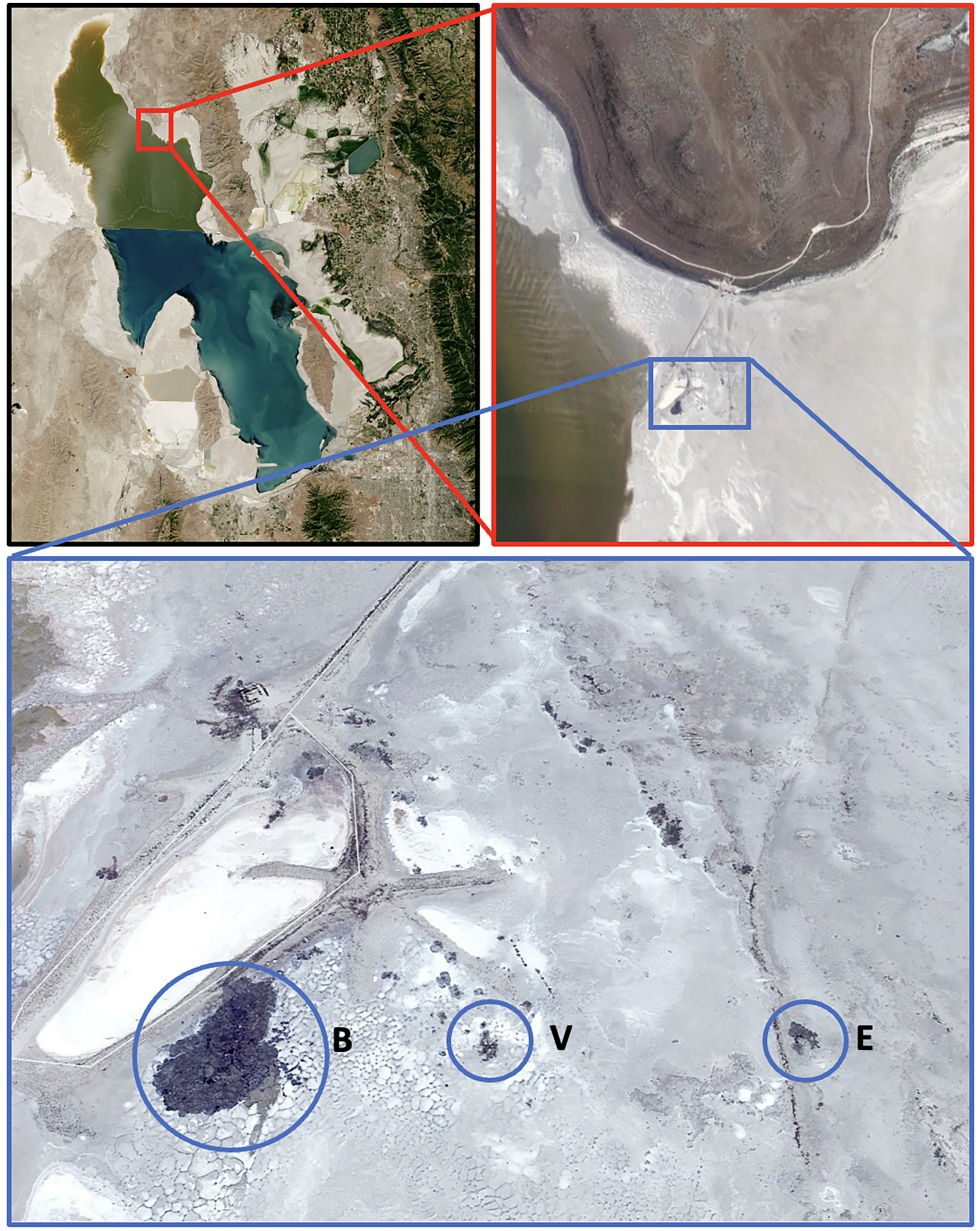
Rozel Point tar seeps on the north shore of Great Salt Lake. The top left panel shows a Google Earth image of Great Salt Lake (2020) with a red box indicating the study area at Rozel point. The top right panel is a magnification of this study area with a blue box to highlight the location of the three seeps we analyzed, which are marked with blue circles on the bottom panel. Our study nomenclature for the seeps are indicated as (B) Big seep, (V) Volcano seep, and (E) Entrapment seep.

Terminal lakes such as Great Salt Lake have been shrinking as water diversions for consumptive uses have increased (Wurtsbaugh et al., 2017; Null and Wurtsbaugh, 2020) and climate change pressures have reduced inflows (Baxter and Butler, 2020). Great Salt Lake’s elevation has dropped so much that almost half the lakebed is exposed, and the pressure on cracks and fissures in the rock under the lakebed has likely changed without the weight of water that used to fill the basin. The Rozel Point tar seeps, along the north shore of Great Salt Lake, are no longer under water and are visible as high molecular weight asphalt oozing from the surface (Figure 1). The spreading tar on the dry shore forms relatively thin layers (less than 10 cm thick) spanning out from the source, and gas bubbles are observed with regularity (Kornhauser et al., 2020).

The Rozel heavy oil reservoir formed during the Oligocene to Pliocene (about 24–1.8 Ma) from organic-rich lake sediments deposited from a prior paleolake that occupied part of the Bonneville Basin (Damsté et al., 1987). Some data suggest even older deposition as cores collected during exploration drilling encountered a volcanic tuff near the bottom of one of the wells, dated at approximately 30 Ma (Oligocene) (Bortz, 1987; Bortz, 2002). About 24 meters under the modern lakebed, the petroleum is in a 0.6–0.9 meter-thick, porous basalt, with associated limestone (Cook et al., 1966; Bortz, 1987; Bortz, 2002). Faults and fractures extend through the rock and facilitate the upward migration of the petroleum (Eardley, 1963). According to geochemistry studies, oil seeping from the ground at Rozel Point likely comes from Tertiary source rock that was deposited in a hypersaline playa (Meissner et al., 1984; Ten Haven et al., 1988). Novel organic sulfur compounds identified in petroleum extracts from this location are consistent with the saline environment model (Damsté et al., 1987). This area was the subject of hydrocarbon exploration from the early 1900s until the 1980s (Eardley, 1963; Hunt and Chidsey, 2002; Gwynn, 2006). The field was abandoned due to the high cost of refining the high molecular weight product and the historic rise in the elevation of Great Salt Lake after several years of unusual snow melt cycles in the 1980s.

Petroleum-enriched sites such as the Rozel Point tar seeps may host a variety of extremophilic life that thrive on the high-molecular weight carbon sources and limitation of some nutrients, including oxygen. Almost a century ago, scientists first isolated bacteria from active oil wells (Bastin et al., 1926), and cultivation has continued to elucidate microbial communities at such locales. These consortia from underground are typically enriched in anaerobic prokaryotes that reduce sulfate or nitrate and/or produce methane (e.g., Magot et al., 2000; Pannekens et al., 2019; Rajbongshi and Gogoi, 2021; Kadnikov et al., 2023). Of course, molecular techniques have further enhanced our understanding of petroleum microbial diversity (e.g., Kim and Crowley, 2007; Lenchi et al., 2013; Kadnikov et al., 2023) and have led to the expectation of thriving communities of both bacteria and archaea even in high molecular weight asphaltic oil. The complex mixture of hydrocarbons and organic compounds in natural petroleum seeps provide many possible substrates for microbial metabolism, and the extremophile consortia are unique to the environmental parameters of a particular site. The high salt in the Great Salt Lake environment should not be an impediment; oil degradation by halophilic bacteria from hypersaline environments is well-documented (e.g., Riis et al., 2003), including at Great Salt Lake (Prince and Prince, 2022).

Early studies of the Rozel Point tar Seeps failed to cultivate any microorganisms, but microbial degradation of petroleum samples was observed in microcosm experiments monitoring the rate of metabolism of labeled hexadecane (Ward and Brock, 1978). Also, phytanes were removed in enrichment cultures, implying biodegradation, which was confirmed in recent work (Prince and Prince, 2022). All of these experiments were done with lake water, modeling the seeps under a higher lake level. This begs the question about metabolism in the Rozel liquid asphalt reservoirs that are no longer under Great Salt Lake brine. Cultivation studies resulted in the isolation of nine genera of haloarchaea and nine genera of bacteria, but no methanogens nor sulfate reducers, likely due to aerobic culture conditions (Haws, 2007). Rozel sediment samples were enriched to select for a community that would degrade the sole carbon source of either benzene or toluene across a range of salinities, from 140 to 290 g/L (Sei and Fathepure, 2009). In this mixed culture, *Gammaproteobacteria* were dominant and implicated in the degradation process. An environmental DNA clone library study from Rozel resulted in a significant proportion of the clones clustered with bacterial species that degrade petroleum (Tazi et al., 2014). All of these studies focused on prokaryotes and are consistent with other findings at hydrocarbon-rich locales (Das and Chandran, 2011), especially those in hypersaline environments (Kadnikov et al., 2023).

There is limited data on fungi at the Rozel Point tar seeps, but this gap in the data is likely because the probes typically used in environmental studies do not illuminate the fungal members of the microbial community, not because these extremophile eukaryotes are not present. Baxter and Zalar (2019) reported cultivating *Aspergillus fumigatus* and *Coniochaeta polymorpha* from Rozel petroleum, and e-DNA spatial studies on Great Salt Lake have illuminated other genera present (Parrott and Baxter, 2024). A broad range of fungi have been noted in other hydrocarbon rich environments and have been a target of the search for bioremediation solutions (Das and Chandran, 2011).

Our study examines the microbial diversity of the Rozel Point tar seeps, analyzing the environmental parameters and consortia at three distinct sampling sites. Given the nature of the viscous asphalt, some of the species identified may be introduced versus endemic microorganisms in the hydrocarbons. In fact, a variety of entrapped animals were recently documented at the Rozel seeps, unable to flee the sticky tar (Kornhauser et al., 2020), which would have contributed to the microbiota. Thus, it stands to reason that the tar might also capture airborne consortia blowing in from the surrounding landscape. This study is then a snapshot of microbial life encounters with the Rozel seeps.

## 2 Materials and methods

### 2.1 Site parameters and field sampling

All sampling took place at the Rozel Point tar seeps on the north shore of Great Salt Lake (Box Elder County, UT, United States). Environmental parameters of the site were recorded over the study period. The surface temperature was assessed with an infrared temperature gun (62 MAX Plus IR Thermometer, Fluke, Everett, WA), and the salinity of any associated surface water was measured with a high salinity refractometer (Model R9600, Reed Instruments, Wilmington, NC). Air temperature data were downloaded from recorded measurements from a nearby weather tower (MesoWest, University of Utah).^1^ Figure 1 and Table 1 describe the location and brief characterization of the three seeps in the study. Samples were taken from these active sites where petroleum was seeping from the ground in the summer of 2019, 2020, and 2021. Samples were collected with sterile spatulas, 10 cm below the surfaces of all three sites, and delivered to sterile 50 ml conical tubes. These were frozen onsite in liquid nitrogen for transport to the lab and then stored at -80°C for later processing. This surface sampling approach may bias the results toward aerobic consortia.

**TABLE 1:** Sampling locations at Rozel Point and site descriptions.

Site name	GPS coordinates	Approximate dimensions (m)	Salinity of associated brine (%)	Field observations
*Big seep*	41°25’49”N 112°39’47”W	374 x 274	20.2 ± 2.1	• Largest seep • Moderate degassing • Moderate surface water
*Volcano seep*	41°25’49”N 112°39’41”W	14 x 9	25.4 ± 2.3	• Prominent degassing elements • Dynamic surface water flows
*Entrapment seep*	41°25’49”N 112°39’35”W	67 x 31	27.7 ± 1.2	• Diverse range of entrapped animals • Limited degassing • Low amount of surface water

Salinity is of the surface water associated with the seep and is reported as an average over sampling periods.

### 2.2 Gas sampling and analysis

Degassing bubbles and inflating areas were captured via motion-activated cameras (20MP Infrared Trail Game Hunting Camera, Cuddleback, Green Bay, Wisconsin). This allowed us to visually observe relative emission of gasses at individual seeps. We conducted direct ground measurements of the flux of methane (CH_4_) and carbon dioxide (CO_2_) (e.g., Etiope et al., 2017) from all three sites described in this manuscript using a closed chamber G2210-i Isotope Analyzer instrument (Picarro, Santa Clara, CA). The data were collected in real time via Bluetooth to the Picarro analyzer and later exported for analysis. We recorded peak gas emission over 10-min intervals for both dry CH_4_ and CO_2_ and averaged the maxima for each seep. As a control, we also conducted measurements of the adjacent oolitic sand sediment, which allowed us to perform *t*-tests and compute significance *p*-values.

### 2.3 DNA extraction from tar samples

Frozen petroleum samples were ground with a sterile ceramic mortar and pestle into a fine soil-like consistency. DNA was extracted from ∼5 mg of each sample using the FastDNA SPIN Kit for Soil (MP Biomedicals, Germany), and we extracted a sample of molecular grade sterile water to provide a blank for later sequencing (Martin, 2022). We quantified the extracted DNA with a NanoDrop microvolume spectrophotometer (ThermoFisher Scientific, Waltham, MA). Peaks were observed at a 230 nm wavelength absorbance, not at 260 nm, the expected wavelength absorbed by DNA. This chromatic shift was apparently associated with contaminants from the petroleum and was rectified with a phenol-chloroform extraction. After this cleaning step, the resulting samples from the microbial community present at each seep contained between 5 and 10 μg DNA. Since ours was a spatial study and not a temporal one, we pooled the DNA from each seep, so each sample that we amplified and sequenced represented microorganism biosignatures over the 3-year collection period.

### 2.4 PCR Amplification, DNA sequencing, and microbial diversity analysis

The DNA samples were transferred to MR DNA (Shallowater, TX, United States)^2^ for gene amplification and to sequence the PCR amplicons using the Illumina MiSeq system. MR DNA amplified the 16S rRNA genes of bacteria and archaea using the 515 forward and reverse primers for the V4 variable region (Walters et al., 2016), the 18S rRNA genes of eukaryotes using EUK1391 forward and reverse primers for the hypervariable region (Rodriguez-Sanchez et al., 2019), and the Internal Transcribed Spacers 1 and 2 of fungi using ITS1-2 forward and reverse primers (Walters et al., 2016; White et al., 1990). The raw and filtered read counts were targeted at 20,000–50,000 raw reads per sample. The DNA sequence raw data was processed to a quality score of Q25, using the MR DNA ribosomal and functional gene analysis pipeline, which began with custom scripts for barcode/primer depletion, size-filtering, and removing sequences with ambiguous base calls or homopolymer runs exceeding 6 bp. Sequences were quality filtered using a maximum expected error threshold of 1.0 and dereplicated. The dereplicated or unique Operational Taxonomic Unit (OTU) sequences were denoised using mothur (v1.x); unique sequences identified with sequencing or PCR point errors were removed, followed by chimera removal, thereby providing a denoised sequence or zOTU. QIIME was used for generating rarefaction curves. The NCBI BLASTn database, along with GreenGenes and Ribosomal Database Project (RDP), were utilized for primary taxonomic classification of the resulting zOTUs. MRDNA provided us with the abundance measures for each OTU represented. We supplied our DNA extraction kit elution buffer as a control for possible contamination of reagents, which allowed us to eliminate signals from any background contaminating DNA. We designed and employed a script^3^ to prune the OTUs relative to the controls, retrieve the taxonomy from open tree of life,^4^ and calculate post-pruning percentages.

For diversity metrics, we focused on the prokaryotic taxa since the eukaryotic species were limited in our data sets, and we had a robust community of bacterial signatures. The bacterial 16S rRNA gene amplicon sequences were demultiplexed and trimmed for quality control to make a feature table of representative sequences and their frequencies using the DADA2 plugin of Qiime2 2025.10 (Caporaso et al., 2010). All samples were trimmed to an equal sampling depth of 22,776 to establish equal feature frequencies across samples and maintain sufficient saturation. Saturation was confirmed by alpha rarefaction analysis for all data sets and all three sampling sites (Supplementary Data 1). Classification of sequences were assigned using the SILVA 138.2 database (Quast et al., 2013; Yilmaz et al., 2014). Qiime2 2025.10 was also used for alpha and beta diversity metrics such as Shannon’s Diversity, Pielou’s evenness, Weighted UniFrac, Unweighted UniFrac, Jaccard’s index, and Bray-Curtis index.

## 3 Results

### 3.1 Field site observations

#### 3.1.1 Environmental parameters

A terminal lake naturally fluctuates in water level, but the elevation of Great Salt Lake has been declining rapidly in the last three decades due to a combination of factors including water diversions (Wurtsbaugh et al., 2017), megadrought (Zhang et al., 2021), and climate change (Baxter and Butler, 2020). As the shorelines have receded at Rozel Point, oozing petroleum from the seeps is evident, consistently emanating from underground. During the period of this study, the shoreline at the site was 1.1–1.3 km from the tar seep area and located in more accessible dry playa. Our team chose three seeps (study names: Big, Volcano, and Entrapment) to analyze based on distinct features that we observed (Table 1).

**FIGURE 2:**
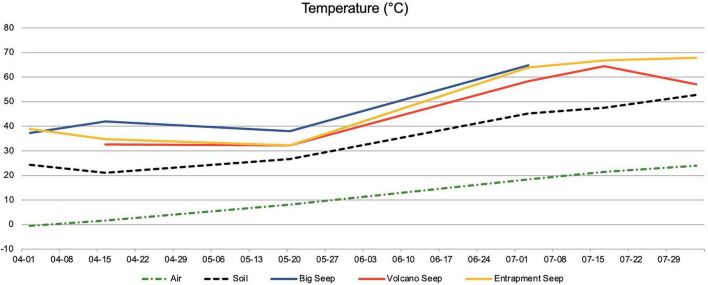
Surface temperature of the three tar seeps in the study compared with average soil and air temperatures in the spring and summer of sampling year 2020. Big seep temperatures are plotted in blue, Volcano in red, and Entrapment in yellow. The air temperature is shown as a green dot-dash line and the soil temperature as a black dashed line.

We measured the temperature differential of the seeps versus the local air and soil. Over one spring and summer (2020), we measured the temperature of each seep around mid-day. We compared these data to the recorded air temperature on the same dates and times and our own measured average soil temperature of the playa surrounding the seeps (Figure 2).

**FIGURE 3:**
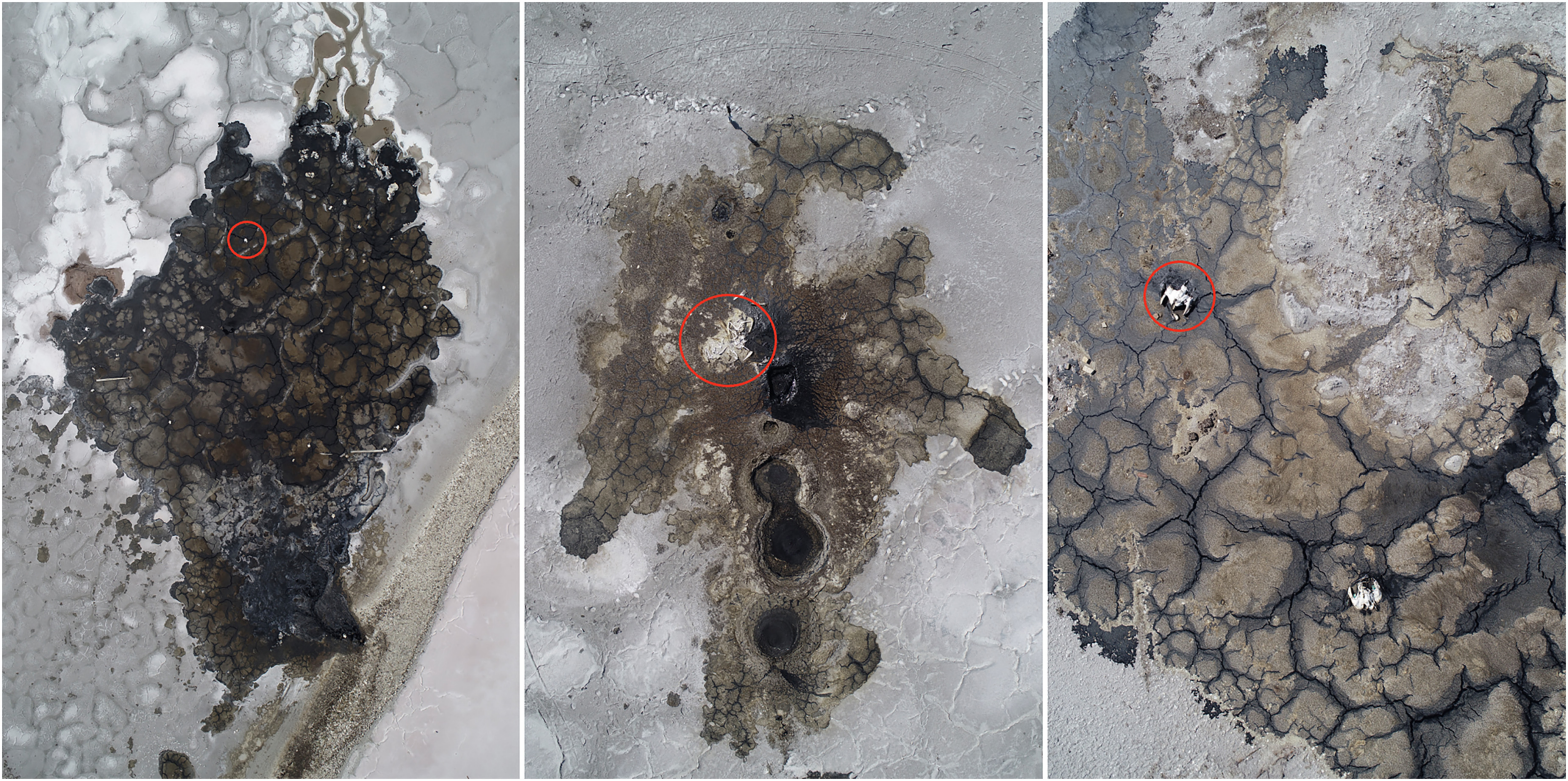
The Rozel tar seeps examined in this study, described in Table 1. Big seep is shown in the left panel, Volcano seep in the middle, and Entrapment seep on the right. The red circles show the remains of pelicans, which can be used for visual scale; American White Pelicans are 127–165 cm long with a wingspan range of 244–290 cm (Cornell Lab of Ornithology, 2025). Drone image credit: Utah Department of Natural Resources.

At near peak solar radiation, the dark colored seeps were all elevated in temperature compared with the high albedo soil and the air temperature. The seeps averaged a temperature 13.9°C higher than the soil measurements and 38°C higher than the air. In the field we observed that elevated temperature resulted in a higher viscosity of the tar, causing more animal entrapments to occur in the summer. All of the seeps are dangerous to the fledglings of a nearby pelican colony on Gunnison Island in the Great Salt Lake north arm (Kijowski et al., 2020), who may mistake the asphalt for water. Figure 3 shows these entrapped birds, which also serve as a visual scale marker for the three seeps under study.

#### 3.1.2 Big seep

In the 1980s, oil companies leased land from the State of Utah in an attempt to pump petroleum from the ground at Rozel Point (Hunt and Chidsey, 2002; Gwynn, 2006). Big seep was one of the drilled sites during this exploration. It is fed more consistently by the reservoir thereby being the largest seep in the area at more than 0.1 km^2^ (Table 1 and Figure 3). This is also the site of measured geothermal activity; a uniform gradient of +65 °C/km was observed from the surface to the bottom of the well at 3.8 km, where the maximum temperature was recorded at 230 °C (Allis et al., 2011). Since Big seep was fed from this warm drill hole, it has a slightly elevated surface temperature relative to the other seeps (Figure 2). In our field site observations, Big seep was a sprawling emergence of asphalt, emanating from a single source. Ground water bubbled up through the tar in shifting locations, forming pockets of surface water.

#### 3.1.3 Volcano seep

**FIGURE 4:**
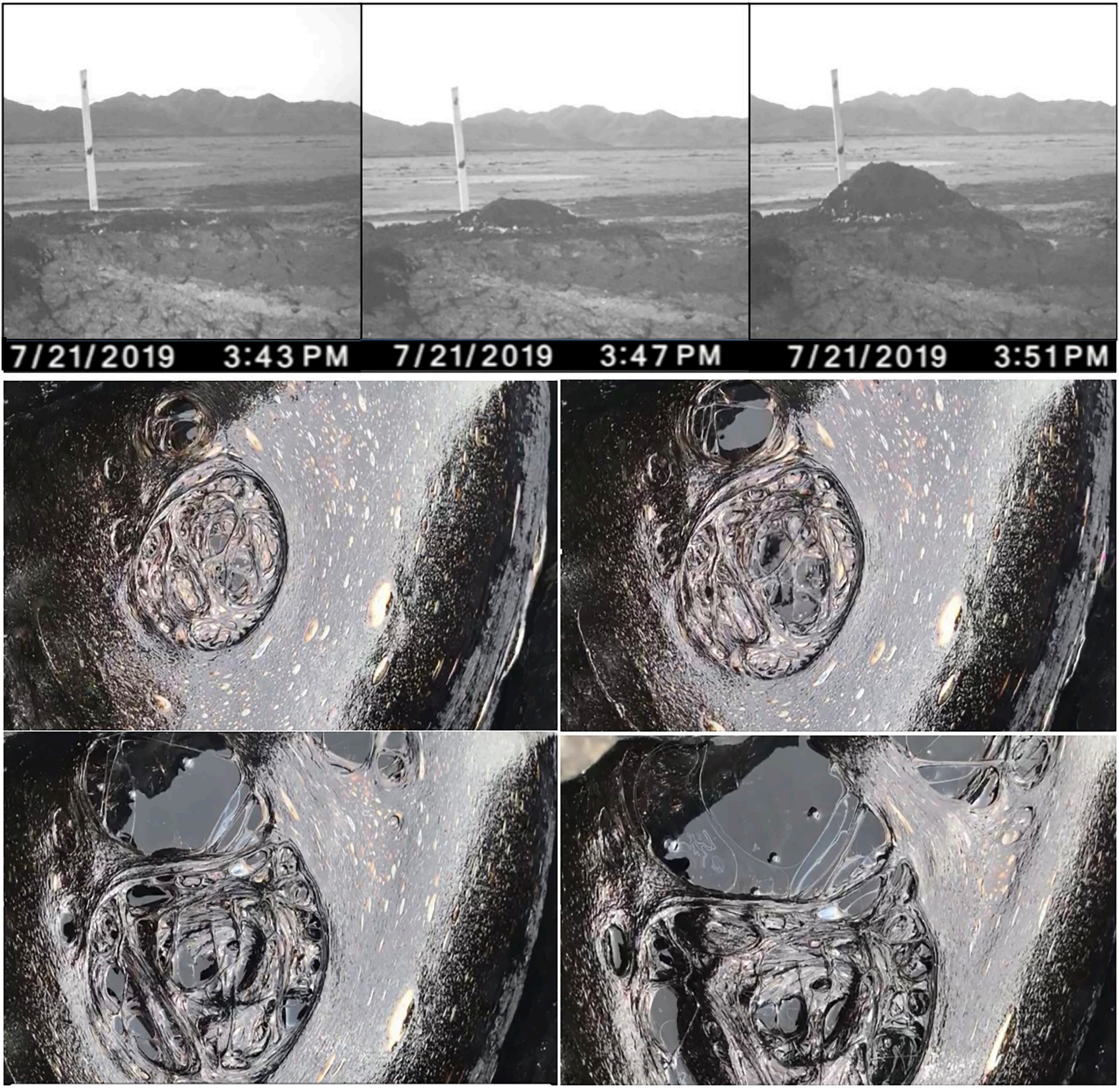
Evidence of gas emissions and ground water intrusion at Volcano Seep. The top panel of motion activated trail camera captures the swelling of petroleum features due to gas emerging from beneath. A meter stick is shown for scale. The bottom panel of gas bubbles and groundwater emerge over a 15 s time frame.

Volcano seep is a natural occurrence, named such because of the similarity in appearance to mud volcanoes which show surface degassing from deep sediments (Dimitrov, 2002). This seep was selected for our microbial diversity study because of the prominence of these degassing elements, which vary in size and number, likely dependent on the temperature and resulting change in viscosity or on the shifting groundwater influx. We observed significant activity at Volcano seep, in the form of frequent bubbles and inflating areas, in real time and also via motion-activated cameras. The top panel of Figure 4 shows escaping gasses pushing the asphalt into a mound over the course of a few minutes. We also captured video of the dynamic Volcano seep as water and gas bubbled on the surface of the tar (Figure 4, bottom panel).

#### 3.1.4 Entrapment seep

**FIGURE 5:**
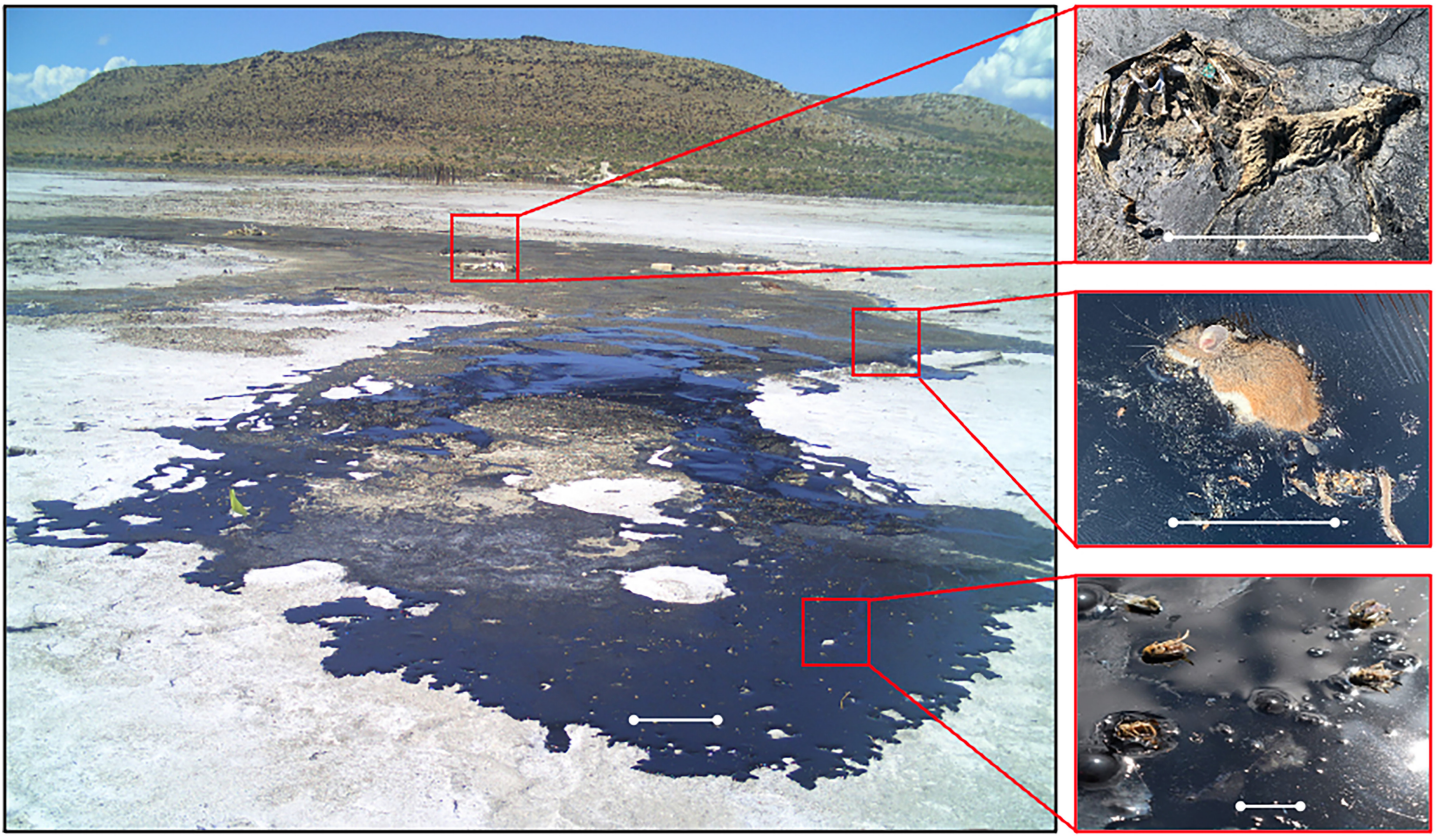
Entrapment Seep emerges and forms a deep puddle of sticky tar which can entrap animals of all sizes (Kornhauser et al., 2020) (white scale bar is 1 m). The top right panel shows a decaying pelican, which we monitored with a trail camera. According to our time lapse recordings, the coyote (whose carcass is beside the pelican in this photograph) came to scavenge the bird, but sat down to rest and got stuck in the tar (white scale bar is 1 m). The middle right panel shows a mouse (white scale bar is 10 cm). According to our trail cameras, we have frequently seen mice running across the cool tar at night when it is less viscous; this one was likely entrapped when the temperatures were higher. The bottom photo shows small flies, likely brine or alkali flies common to Great Salt Lake (white scale bar is 1 cm). The animal life would add to the microbiome of this seep.

Entrapment seep, also natural, is a shallow and dense mat of tar with limited water intrusion. Surface water was higher in salinity than the other seeps (Table 1), suggesting more evaporation and concentration. Here, the high viscosity material has collected not only pelicans that we observed at all seeps, but a wide variety of entrapped animals, from smaller birds to mammals to insects. The unlucky animals were stuck here over the last decade when the seeps have been exposed by the retreating Great Salt Lake shoreline (Kornhauser et al., 2020). Entrapment seep was chosen as a study site because of the immense number of carcasses associated with the tar and how that might impact the microbiology at Rozel Point (Figure 5).

### 3.2 Gas analysis

Biodegradation of hydrocarbons by prokaryotes and fungi can release carbon dioxide (CO_2_) or methane (CH_4_) (Larter et al., 2005; Das and Chandran, 2011). We suspected that the degassing features observed at the field site (Table 1) might be evidence of active microbial activities, and we wanted to understand the composition of gasses released. At all three seeps, we detected a strong CO_2_ signal, but the values were consistent with expected atmospheric levels (Humble et al., 2026). The averaged CO_2_ measurements were 430 ± 6 ppm for Big seep, 421 ± 18 ppm for Volcano, and 469 ± 98 ppm for Entrapment. In comparison with atmospheric measurements and our sediment control (427.09 ± 5.71 ppm), we do not see evidence of values elevated by microbial metabolism. CH_4_ signals were received in pulses, and we averaged the maxima of the peaks. The sediment control CH_4_ measurement was 2.64 ± 0.61 ppm. At Big seep, where we observed small bubbles, limited CH_4_ was detected, 8.15 ± 0.08 (Table 2). Whereas at Volcano seep, where we frequently observed emerging gas bubbles and swelling of the surface tar (Figure 4), and at Entrapment seep, the peak CH_4_ emission measurement was elevated, 194.76 ± 11.97 and 232.96 ± 53.49, respectively, suggesting methanogenesis at these sites. No other gasses were identified. We ran a one-way ANOVA analysis to compare the differences between the three seeps. The test on the low CH_4_ measurement from Big seep indicated that it was significantly different and distinct from the other two seeps. However, Volcano and Entrapment seep CH_4_ ppm values are not statistically different, given the overlap considering standard deviations, and these seeps represent hotspots of CH_4_ release. This spatial heterogeneity and pulses in the CH_4_ signals received by the gas analyzer may be due to the observed ebullition, typical with subsurface methanogenesis (Hermans et al., 2024).

**TABLE 2:** Methane emissions from the Rozel tar seeps.

Site name	CH_4_ (ppm)	*t*-test	Significance (*p*)
*Big seep*	8.15 ± 0.08	15.51	0.0036
*Volcano seep*	194.76 ± 11.97	27.76	0.0013
*Entrapment seep*	232.96 ± 53.49	7.46	0.0175
*Control*	2.64 ± 0.61	–	–

The mean and standard deviation (SD) are reported for the peak maxima for methane (CH_4_) in parts per million (ppm) at each location. Measurements from the adjacent oolitic sediment are included and used as a control to run the *t*-test and compute significance.

### 3.3 Microbial diversity at the Rozel Point tar seeps

The observations of warm temperatures (Figure 2), degassing elements (Figure 4), entrapped animals (Figure 5), and released CH_4_ (Table 2) suggests that the Rozel seeps may feature a unique set of microbial metabolisms. The key hypothesis driving this work was that there is a vibrant microbial community in the Rozel tar seeps that includes bacteria, archaea, and fungi, and some of these species would be able to use the high molecular weight hydrocarbons as a carbon source. Below we present taxa abundances, and we compare the findings across the three sampled seeps. This spatial approach allows us to consider the environmental conditions that are distinct at each site and also to highlight any common taxa.

#### 3.3.1 Archaea

**FIGURE 6:**
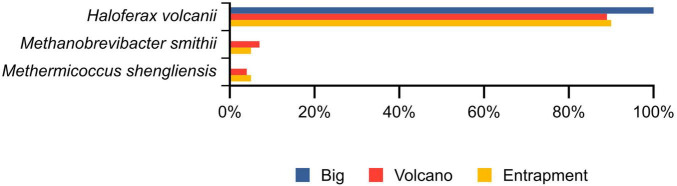
Best matched archaeal species by relative abundance in the Rozel seeps. Blue represents amplicons from Big seep, red from Volcano, and yellow from Entrapment.

We surveyed prokaryotes at the three seeps with 16S rRNA amplification. Since a broad diversity of haloarchaea dominate the Great Salt Lake north arm brine (e.g., Tazi et al., 2014; Almeida-Dalmet et al., 2015; Almeida-Dalmet and Baxter, 2020) and minerals (Kemp et al., 2018; Bayles et al., 2020; Martinez-Koury et al., 2025), we predicted that these taxa would be prevalent in the nearby Rozel seeps. However, only one haloarchaea species, related to *Haloferax volcanii*, was observed, but it was in high abundance (Figure 6). The amplicon sequences also included two other archaea taxa, best matched to *Methermicoccus shengliensis* and *Methanobrevibacter smithii* in Volcano and Entrapment seeps. This limited diversity, of only three archaeal species, was unexpected.

#### 3.3.2 Bacteria

**FIGURE 7:**
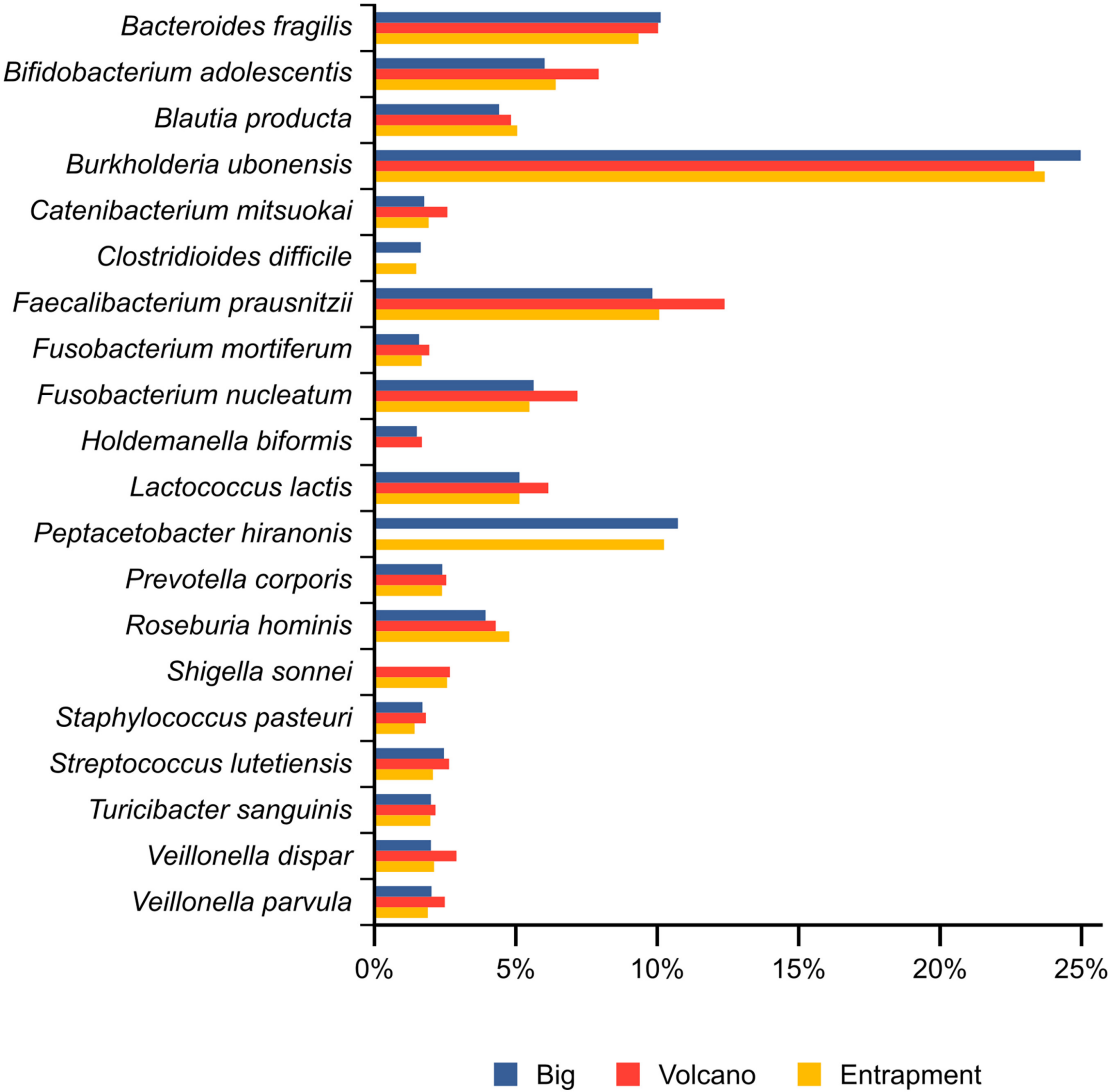
Best matched bacterial species by relative abundance in the Rozel seeps. Represented here are the species whose abundance measures exceeded 1% in the bacterial sequences. Blue represents amplicons from Big seep, red from Volcano, and yellow from Entrapment.

The most abundant and diverse taxa were observed in the bacterial OTUs; the data indicate signatures of more than 300 species (Supplementary Data 2). Almost half (48%) of the species observed were classified under the phylum *Bacillota* and almost a third (27%) are *Pseudomonadota* (formerly *Proteobacteria*). Other significant phyla include *Bacteroidota* (9%), *Actinomycetota* (8%), and *Fusobacteriota* (5%). We plotted the most predominant OTUs present, binning at species level to compare their relative abundance (Figure 7). We observed similar species and abundance patterns across all three seeps with only a few exceptions. The *Peptoclostridium* and *Peptacetobacter* species were missing from Volcano seep, and the *Holdemanella* species was missing from Entrapment seep. The most common and ubiquitous species identified was best matched to *Burkholderia ubonensis*. In our data analysis, we realized that all other OTUs correlate with species that are pathogens or normal microbiome of mammals.

Despite the similar relative abundances in the most abundant bacteria, principal coordinates analysis (PCoA) done on 16S rRNA gene sequences based on dissimilarity of species relative abundance (Bray-Curtis) and relative abundance considering phylogenetic distance (Weighted UniFrac distance) showed clear separation between all three seeps indicating substantial differences in microbial composition and phylogenetic relatedness (Supplementary Data 3). Community similarity based on species absence and presence (Jaccard’s Index) and absence and presence considering phylogenetic distance (Unweighted Unifrac distance) also showed variance between all three sites (Jaccard, 1908; Bray and Curtis, 1957; Lozupone et al., 2007). Alpha diversity analyses of the prokaryotic community of each seep were carried out using the 16S rRNA data. Shannon diversity and Pielou’s evenness indices showed similar values in the diversity metrics and species distribution between all sites; all sites had Pielou values higher than 0.9, indicating strong evenness (Table 3). Group significance of these diversity metrics could not be measured since samples from each site were pooled into single replicates. Since the PCoA analyses were done on all 16S rRNA gene data, this includes archaea sequences. However, before trimming, archaea only accounted for 0.40–0.61% of the 16S rRNA data (Supplementary Data 3).

**TABLE 3:** Alpha diversity analyses showing Shannon diversity and Pielou’s evenness indices of the prokaryotic community of each sampling location.

Site name	Shannon diversity	Pielou evenness
*Big seep*	6.670	0.913
*Volcano seep*	6.232	0.914
*Entrapment seep*	6.646	0.909

#### 3.3.3 Fungi

**FIGURE 8:**
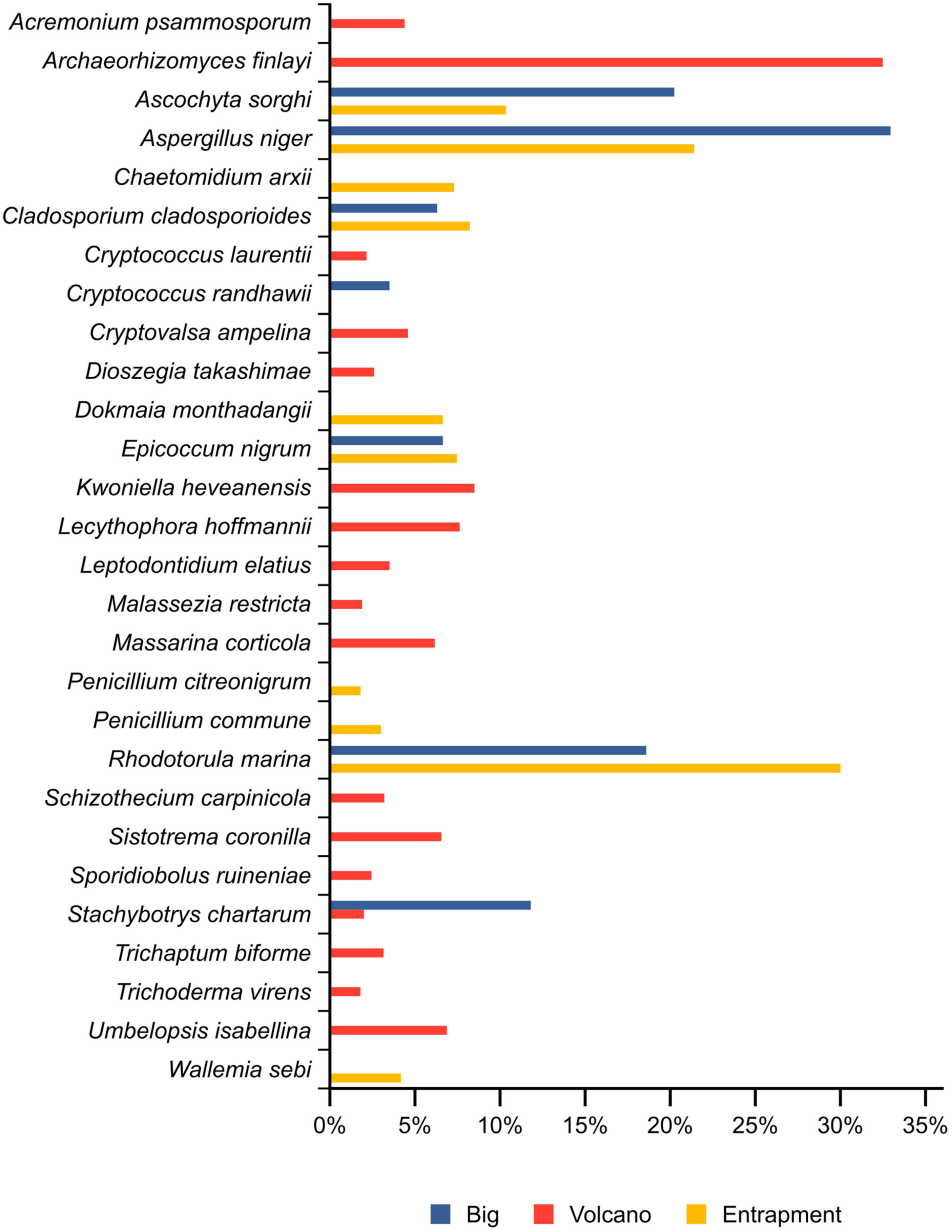
Best matched fungal species by abundance in the Rozel seeps. The taxa indicated are present at an abundance greater than 1%. Blue represents amplicons from Big seep, red from Volcano, and yellow from Entrapment.

Recently we (DLP and BKB) reviewed fungi studies around a number of niches at Great Salt Lake for a spatial study (Parrott and Baxter, 2024), and we incorporated the Rozel tar seep OTU dataset in our review. Here we analyze the details sorted by abundance and seep (Figure 8). Among the predominant fungi, none of them occur at all three sampled seeps. In the analysis, we noted several categories of fungi based on niche: those associated with petroleum, animals, plants, or brine, which we explore in the discussion below.

In separate amplifications, we used 18S rRNA primers for eukaryotes (data not shown), but the results included only fungal OTUs, which are better represented by the more specific ITS primer amplicons (Figure 8) as they give more reliable abundance data (White et al., 1990).

## 4 Discussion

As modern Great Salt Lake recedes to historically low elevations, petroleum from deep underground is now accessible at the Rozel tar seeps. We chose distinct sampling sites that had different features in an effort to understand the microbial diversity. The most surprising theme in our data is just how similar these three sites are. For example, the bacteria and archaea profiles at each seep (Figures 6, 7) are not only similar in composition, but the particular species are also similar in abundance measures. The fungi (Figure 8) break these rules, however, but given their robust dispersal methods (Golan and Pringle, 2017; Chaudhary et al., 2022), encounters of spores with the seeps may be more sporadic and random. The caveat in looking at biosignatures like DNA is that some species may be alive and actively metabolizing, others might be fossilized and preserved in the seeps, and still others could just be introduced by wind or water. Our future studies at this site will add other biochemical approaches beyond eDNA signals, such lipid biomarker analyses or metabolomics, to verify the presence of cells and their metabolisms. The Rozel tar seeps are likely biological reservoirs for both endemic microorganisms and introduced biosignatures over time.

Any living microorganisms in the Rozel seeps may inhabit not the tar itself but the associated water droplets or at the oil-water transition zone as observed in other hydrocarbon rich sites (reviewed in Pannekens et al., 2019). The amount of water at our site may be important; oil reservoirs with a higher water content (40–60%) enriched bacterial diversity over the low water content sites (1–5%) by 2.6-fold (Korenblum et al., 2012). Although the Rozel seeps are no longer under water, hydration occurs through seasonal precipitation and year-round groundwater intrusion (Figure 4), and the water phase within the petroleum may exist as small pockets and may be crucial for microorganisms dependent on the rich carbon sources of the tar (Meckenstock et al., 2014; Pannekens et al., 2019). There is greater fungal diversity at Big and Volcano seeps (Figure 8) where abundant water was observed (Table 1), but this did not impact the microbial diversity of the bacteria and archaea. The amount of surface water features may not track with the water composition beneath the surface.

The diversity metric analyses (Supplementary Data 3) suggest the bacteria communities between seeps differ in species presence and absence, relative abundance, and phylogenetics. Since the relative abundance of predominant bacteria between all three seeps are similar, it is possible the community differences are driven mostly by variation among the less abundant taxa of our samples (Supplementary Data 2).

### 4.1 Limited halophilic taxa

The hypersaline north arm of Great Salt Lake is enriched with halophilic archaea (e.g. Tazi et al., 2014; Almeida-Dalmet et al., 2015), bacteria (Almeida-Dalmet and Baxter, 2020), and fungi (Parrott and Baxter, 2024). Given the proximity of the Rozel seeps to the lake and the fact they were covered by the lake water until recently, we expected the halophilic microorganisms associated with the lake and its minerals to be introduced to the tar that emanates from below this playa. However, signatures of common hypersaline prokaryotes were rare; the bacteria typically associated with salty systems were absent and the haloarchaea were limited to a single species. We only observed a match for *Haloferax volcanii* in our archaeal data set, but it represented an abundance of more than 85% of archaea for all sampled seeps (Figure 6). This species was previously isolated from soils infused with petroleum and brine (Emerson et al., 1994), suggesting that *Haloferax* species are well suited for this habitat.

Among the fungal components of the Rozel tar community, *Wallemia sebi* was an expected matched species since it had been previously cultured from south arm lake water (Baxter and Zalar, 2019), and its spores are air-disseminated (Zeng et al., 2004). This genus is considered one of the most xerophilic eukaryotic life forms, surviving in many low water activity habitats on Earth such as salt lakes and minerals (Zalar et al., 2005; Jančič et al., 2015). We also observed *Acremonium psammosporum* among our OTUs, and members of this marine-borne genus (Jones et al., 2009) have been found in Great Salt Lake brine and salt crystals (Baxter and Zalar, 2019) and at other thalassohaline lakes (Grum-Grzhimaylo et al., 2018). However, both *Wallemia* and *Acremonium* were low in abundance (4%) and each only found at a single seep.

Since the associated brine at each tar seep had similar salinities to the nearby Great Salt Lake north arm (Table 1) and would support the growth of halophiles, we hypothesize that the low abundance of halophilic taxa is likely due to the microbial interface with the hydrocarbons. One possibility is that the interplay between hydrophilic salts versus hydrophobic tar would put stress on the structure of the cell membranes, even in an osmophile (McGenity, 2010). Another is the possibility of toxic effects of the hydrocarbons although a number of halophilic archaea and bacteria have strategies to overcome this (Martínez-Espinosa, 2024). When taking samples, we targeted the tar and not the brine, thus the lack of halophile signals may simply be due to sampling bias.

### 4.2 Petroleum degrading consortia

On the shores of Great Salt Lake, the Rozel tar seep hydrocarbons provide organic substrates, and sometimes a sole carbon source for microorganisms in this extreme environment (Prince and Prince, 2022). Universal microbial strategies, with common enzymes for degrading hydrocarbons, exist across all domains of life, and this biodegradation can process the polycyclic aromatic hydrocarbons (PAHs) and release CO_2_ or CH_4_ (Larter et al., 2005; Das and Chandran, 2011; Prince et al., 2019). We observed degassing elements at the seeps (Table 1 and Figure 4), and our gas analysis confirms CH_4_ emissions consistent with these microbial activities (Table 2).

As discussed above (section 4.1), the most abundant archaeal species in our amplicons is a halophile, *Haloferax volcanii* (Figure 6), which lives at salt saturation, but it also degrades PAHs (Al-Mailem et al., 2010; Bonfa et al., 2011). The other two archaeal species are methanogens. At Big seep, where the peak CH_4_ measurement was marginal, we detected no methanogenic taxa. However, in both Volcano and Entrapment seeps, where the CH_4_ emissions were robust (Table 2), we observed biosignatures for *Methanobrevibacter smithii* (4–5% abundance) and *Methermicoccus shengliensis* (5–7% abundance) (Figure 6). *Methermicoccus shengliensis* is an anaerobic, thermophilic archaea that degrades a wide variety of organic compounds to release methane and carbon dioxide during methoxydotrophic methanogenesis (Mayumi et al., 2016; Khomyakova and Slobodkin, 2023). Finding the signature of this taxa in natural tar seeps is consistent with the species being previously isolated from formation water of oil deposits in Asia (Cheng et al., 2007). On the other hand, *Methanobrevibacter smithii* is a methanogen associated with animal digestive tracts, and it is probably not metabolizing the tar (Guindo et al., 2020).

The bacterial signatures were our most diverse data set, but only one matched species hit, *Burkholderia ubonensis* (Figure 7), has potential petroleum-degrading capabilities (Price et al., 2017) and is known for doing methylphosphonate demethylation (Li et al., 2023). *Burkholderia* species are common inhabitants of soil and water that can metabolize a large range of organics (Cupples, 2024). Recently, *Burkholderia* was discovered in Russian oil reservoirs with high saline formation water (Kadnikov et al., 2023), reflecting the high salinity of the lake water near our sampling site. Most of the prokaryotes we identified through sequencing are not known to be associated with petroleum environments, and we assume that they are there incidentally, However, petroleum-degrading fungi may use bacterial metabolites (Rezaei and Moghimi, 2024). This suggests symbiotic relationships could exist between the fungi and prokaryotes in the Rozel seeps endemic communities

We found signals of fungal taxa (Figure 8) that can utilize petroleum as an energy source, the most abundant being *Aspergillus niger* (33 and 21% at two seeps) and *Rhodotorula marina*, (19 and 30% at two seeps). *A. niger* is a ubiquitous fungal species observed at other petroleum-rich sites, which makes lipase and can degrade even high molecular weight tar such as we find at Rozel (Zafra et al., 2014; Mauti et al., 2016; Barnes et al., 2018; Nayak et al., 2020). Some species of marine *Rhodotorula* have been found to degrade PAHs (Shailubhai et al., 1984; Lin et al., 2022), so it is possible *Rhodotorula marina* also has this capability. Less abundant fungi identified in our data that may degrade hydrocarbons include *Cladosporium cladosporioides* (Bakri, 2022; Birolli et al., 2018), *Penicillium citrinum* (Birolli et al., 2018), *Penicillium commune* (Esmaeili and Sadeghi, 2014), *Epicoccum nigrum* (Queissada et al., 2013), and *Umbelopsis isabellina* (Janicki et al., 2018).

Petroleum-degrading microorganisms have been studied as potential bioremediators in contaminated sediment; not only does the water content of the site impact their metabolic activity, but also the temperature, viscosity, and oxygen availability (Van Hamme, 2004; Das and Chandran, 2011). Also, the biodegradation processes are located to a hydrocarbon-water transition zone (Meckenstock et al., 2014). Perhaps, these factors are optimal at the Rozel tar seeps since the tar appears to have a rich fungal species diversity of taxa (alongside some prokaryotes) capable of utilizing the available PAHs.

### 4.3 Introduced biosignatures

The Rozel seeps form sticky mats of tar that are viscous, and at the Goldilocks temperature (not too hot to be flowing nor too cold to be solid), this petroleum ooze can capture animals or plant matter. Previous work at these seeps demonstrated entrapment of animals (Kornhauser et al., 2020), and another spatial survey demonstrated the presence of fungi associated with both animals and plants (Parrott and Baxter, 2024). In our data set, all but one of our bacteria signatures match species associated with animals, and the majority of fungi that we observed are associated with plants (Figures 7, 8). There are no animals nor plants inhabiting the tar, suggesting that the seeps are capturing genetic signals from the sagebrush steppe and ranches that surround the Rozel site. Aerial transport on dust is a likely microbial dispersal mechanism in this desert ecosystem prone to dust events (Kellogg and Griffin, 2006). Dust particles can form a bridge between systems, depositing microbiota downwind.

#### 4.3.1 Animal encounters impact the tar microbiome

Microbiota may be introduced when animals are entrapped in the tar, and their corpses decay over time (Figures 3, 5). Also, Rozel Point is surrounded by ranches that raise cattle and horses, and the direction of surface water runoff would be toward the terminal lake and the Rozel seeps. In our bacterial data set, we noted microorganisms associated with dairy cow infections, *Staphylococcus pasteuri* (Savini et al., 2009) and *Streptococcus lutetiensis* (Chen et al., 2021) as well as a species commonly found in cow milk, *Lactococcus lactis* (Sanders et al., 1999). We also found evidence of bacteria that are likely originated in the mouths of mammals: *Veillonella parvula* is associated with dental plaque (Liu et al., 2020), and both *Fusobacterium mortiferum* and *Fusobacterium nucleatum* are found in the horse oral microbiome (Citron, 2002).

The water inflows toward Great Salt Lake would also bring microorganisms from the excrement from the ranchland animals. We discovered bacterial signatures for the following species that are associated with mammalian intestinal microbiome or enteric pathogens: *Bacteroides fragilis* (Sears, 2001), *Bifidobacterium adolescentis* (Duranti et al., 2020), *Blautia producta* (Liu et al., 2021), *Catenibacterium mitsuokai* (Kageyama and Benno, 2000), *Faecalibacterium prausnitzii* (Miquel et al., 2013), *Clostridioides difficile* (Hernández et al., 2019), *Peptacetobacter hiranonis* (Hanifeh et al., 2024), *Prevotella corporis* (Tett et al., 2021), *Roseburia hominis* (Patterson et al., 2017), *Shigella sonnei* (Torraca et al., 2020), *Turicibacter sanguinis* (Cuív et al., 2011), and *Veillonella dispar* (Xu et al., 2023). In addition to the bacteria, one archaeal taxa matched a species associated with animal digestive tracts, *Methanobrevibacter smithii* (a methanogen discussed above) (Guindo et al., 2020).

This study cannot differentiate between microorganism signatures introduced from ranchland runoff versus decaying animals entrapped in the Rozel seeps. Also, in the case of some species signals observed, it may be hard to determine if they are from microbiota associated with plants instead of animals. For example, there are a few fungal signatures that represent species associated with plants (see below) but can facultatively inhabit mammals. *Malassezia restricta* is from a family of yeasts that can be either commensal or pathogenic with animal hosts (Torres et al., 2020) while *Stachybotrys chartarum* (Dyląg et al., 2022) and *Trichoderma virens* (Kredics et al., 2014) can be mammalian pathogens. Of course, spores from fungi associated with animals (or plants) of the surrounding steppe could become entrapped in the tar after arriving by wind instead of water or animal intruders (Kellogg and Griffin, 2006).

#### 4.3.2 Plant-associated fungi were abundant in the seeps

In our fungi data (Figure 8) two of the most abundant fungal species, *Archaeorhizomyces finlayi* (32%) and *Ascochyta sorghi* (10–20%) are plant-associated. *A. finlayi* is associated with roots of conifers and rhizosphere soils and occur on all continents, except Antarctica, and in most terrestrial biomes and *Ascochyta sorghi* is a grass pathogen. The exposed playa surrounding the Rozel seeps is almost entirely devoid of plant life, save for a few sparsely scattered *Salicornia* spp. established near the access roadways; the e-DNA signatures of numerous plant-associated fungal species are likely introduced by wind. Sticky tar could collect airborne spores or plant material trapped on birds or other animals.

Some of these introduced species are endemic to Great Salt Lake and the surroundings. Based on our previous research, *Penicillium citreonigrum* and *Penicillium commune* signatures were revealed in the shoreline root microbiomes and soils around GSL plants (Parrott and Baxter, 2024). These species are implicated in plant biomass decomposition and carbon and nitrogen cycling in arid landscapes (Kuypers et al., 2018; Egidi et al., 2019). *Cladosporium cladosporioides* (6% in Big and 8% in Entrapment) closely matched a species previously isolated from Great Salt Lake water and minerals (Baxter and Zalar, 2019). Another species in the seeps, *Lecythophora hoffmannii* (8% in Volcano), is implicated in plant biomass decomposition, carbon and nitrogen cycling in arid ecosystems, and virulence in Utah desert plants (Challacombe et al., 2019). This genus is associated with the nearby north warm water (Baxter and Zalar, 2019; De Leon et al., 2023), suggesting that it was introduced from the landscape.

Many other fungal species associated with plants are present at low levels in our data: *Dokmaia monthadangii*, a plant leaf decomposer (Promputtha et al., 2003); *Massarina corticola*, the genus of which contains saprophytes and endophytes of woody plants with a few species that are plant pathogens (Abdel-Wahab et al., 2007); *Leptodontidium elatius*, plant pathogen (Belding et al., 2000.); *Chaetomidium arxii*, rotting plant materials and soils (Wang et al., 2022); *Schizothecium carpinicola*, agriculture soil microbiome (Behnke et al., 2021); *Cryptovalsa ampelina* grapevine, plant pathogen (Luque et al., 2006); *Sistotrema coronilla* eucalyptus, plant associated (Barreto et al., 2023); *Trichaptum biforme* wood decay fungi (Mukhin et al., 2023); *Sporidiobolus ruineniae* plant leaf associated (Kanpiengjai et al., 2016); *Dioszegia takashimae* isolated from plant leaves, roots or soil (Takashima and Nakase, 2001); *Cryptococcus laurentii* and *Cryptococcus randhawii* previously found in Great Salt Lake soils (Parrott and Baxter, 2024).

### 4.4 Sampling depth may impact community detected

Most PAH biodegradation requires anaerobic conditions (Larter et al., 2005), but the most *efficient* enzymatic degradation of petroleum by microorganisms occurs in aerobic conditions (Das and Chandran, 2011). We extracted DNA from surface samples at the Rozel tar seeps and thus selected for more aerobic communities, which is apparent in our results. If we sampled below ground, we would expect to identify more anaerobic strains, such as PAH-degrading sulfate reducers (Rajbongshi and Gogoi, 2021), which were absent in our data set. Sei and Fathepure (2009) cultured bacteria from the Rozel tar seeps by inoculating samples in PAH-enriched media, resulting in several cultivated strains of *Gammaproteobacteria* that could utilize the substrate. The significance of the *Gammaproteobacteria* phylum at petroleum sites was underscored by its predominance of e-DNA signatures at sites such as Brazilian oil fields (Korenblum et al., 2012) and at the La Brea tar pits (Kim and Crowley, 2007; Baquiran, 2010). We detected *Gammaproteobacteria* signals, but they were very low in abundance (well below 1%) and thus pruned from the bacterial data shown in Figure 8 (see Supplementary Data 2 for taxa).

Also, given the underground geothermal sources (Allis et al., 2011), thermophilic taxa could be present if we accessed deeper core samples. Subsurface sampling and extraction from deeps pits at Rancho La Brea revealed a rich diversity of archaea (Kim and Crowley, 2007), which were not found in our data. Our shallow surface sampling retrieved samples that were warmer than air temperatures due to solar radiation (Figure 2), but there was no direct warming apparent from geothermal sub-surface temperatures (Allis et al., 2011). If the Rozel seeps are drilled for asphalt production in the future, a vertical transect experiment may be possible, but under current circumstances, this would be a challenging experiment in this extreme environment.

### 4.5 Astrobiology implications

One significant feature of hydrocarbon seeps in the astrobiology context is that their robust organic compounds can be synthesized from primary carbon sources, for example, carbon monoxide (Glein and Zolotov, 2020). In addition, early Earth petroleum sites may have been composed of extraterrestrial material from carbonaceous meteoritic infalls (Huang et al., 2005), providing prebiotic hydrocarbon sources for the origin and evolution of life on Earth. Beyond supporting abiogenesis, rich organic environments in the universe may also represent microbial habitat as the Rozel seeps do at Great Salt Lake.

The search for possible habitats for microbial life on other space bodies is supported by studies on Earth’s remarkable environments (Schulze-Makuch et al., 2011), and the Rozel tar seeps can serve as an analog for other petroleum reservoirs in our Solar System. The high molecular weight hydrocarbons at Rozel have the highest sulfur content reported for Earth’s sources, up to 14 % (Damsté et al., 1987; Katasonova et al., 2021). Such organic rich chemistry also exists on Saturn’s largest moon, Titan, where the Cassini mission mapped hydrocarbon pools on the surface (Elachi et al., 2006; Stofan et al., 2007); water ice and transient liquid water may exist there as well (e.g. Neish et al., 2006), suggesting interfaces where water and hydrocarbons meet. Titan has a nitrogen rich atmosphere with limited oxygen, allowing carbon to exist primarily in its reduced state, and this methane rains onto Titan’s surface to form organic lakes (Niemann et al., 2010; Hayes, 2016). This surface methane interacts with other chemistry, such as sulfur in the atmosphere, to generate new organic material over a timeline, meaning that future analyses could establish a geologic record over time of these hydrocarbon seeps (Glein and Zolotov, 2020). Titan’s hydrocarbon lakes are a potential (extinct or extant) habitat for microbial communities in organics-water microinterfaces, and thus a biosignature target of interest (Neish et al., 2018).

The insights from our work at Rozel underpin hypotheses regarding biosignature searches on space bodies such as Titan. Since seeps may capture the biosignatures of the local environment and not only what is endemic to the tar (section 4.3), we would hypothesize that hydrocarbon pools on Titan would also collect biosignatures from both endogenous and exogenous sources. Considering the possibility of a geologic record of carbon compounds in a Titan methane lake, multi-marker biosignature detection methods could separate endogenous and exogenous biosignatures based on timelines of introduction. However, one major distinction between Rozel and Titan is temperature, which is estimated to range from -180 to –170°C. on Saturn’s moon (Jennings et al., 2016). We hypothesize that the warmer temperatures at Rozel (Figure 2) contribute to the viscous nature of these tar seeps, leading to entrapment of airborne microorganisms, fungal spores, plant matter, and animals. It follows that we would predict a higher abundance of endogenous biosignatures in the much colder, and less viscous, Titan hydrocarbon lakes.

Remote sensing methods do not have the spectral resolution for biosignature exploration on Titan, which argues for in-situ approaches (Neish et al., 2018). The Huygens probe did land on the Titan’s surface (Lebreton et al., 2005) and measured the first in-situ data on Titan’s atmospheric chemistry and composition (Fulchignoni et al., 2005; Niemann et al., 2005). Although it was equipped with a gas chromatograph mass spectrometer (GCMS) for analyzing the atmosphere, it was not designed for biomolecule exploration on the surface. Targeting biosignature experiments on Titan in the future, researchers and space agencies would require a lander, equipped with instruments designed to identify biomolecules in the hydrocarbon lakes and ice-water environments. Specialized GCMS instruments able to analyze DNA/RNA, amino acids, lipids, and other biological molecules (in a single sample) should be coupled with processing tools such as chiral columns and derivatization agents that will enhance volatilization (reviewed in Neish et al., 2018). These multi-marker approaches are also critical to sort out abiotic versus more complex biotic sources of the compounds. If microbial life flourished in surface petroleum reservoirs on Titan, analogous to the Rozel tar seeps, these biosignature traps would be invaluable for future astrobiology explorations.

## Data Availability

The metagenomic data presented in the study are deposited in the NCBI GenBank Sequence Read Archive (SRA) database repository, accession number PRJNA1147725. All other data or methods are available upon request and are maintained and stored through Great Salt Lake Institute at Westminster University.

## Footnotes

1
https://mesowest.utah.edu

2
www.mrdnalab.com

3
https://github.com/avabaxter/process-otu-data

4
https://tree.opentreeoflife.org/opentree/argus/opentree16.1@ott93302
