# Supplementary material for: Biosignature reservoirs in the Rozel Point tar seeps at Great Salt Lake, Utah
Source: Front Microbiol. 2026 Aug 3;17:1832944. doi: 10.3389/fmicb.2026.1832944 (PMC13479432; doi:10.3389/fmicb.2026.1832944)
Supplement: Supplementary file 1 [file Data_Sheet_1.pdf]

Martin/Sanchez et al. Biosignature Reservoirs in the Rozel Point Tar Seeps
Supplemental Data 1

Alpha rarefaction curves of 16S rRNA (a), 18S rRNA (b), and Internal Transcribed Spacers 1 and 2 sequences (c). Only 16S rRNA data was used for PCoA analyses because of the significant number of prokaryotic species.

(a)

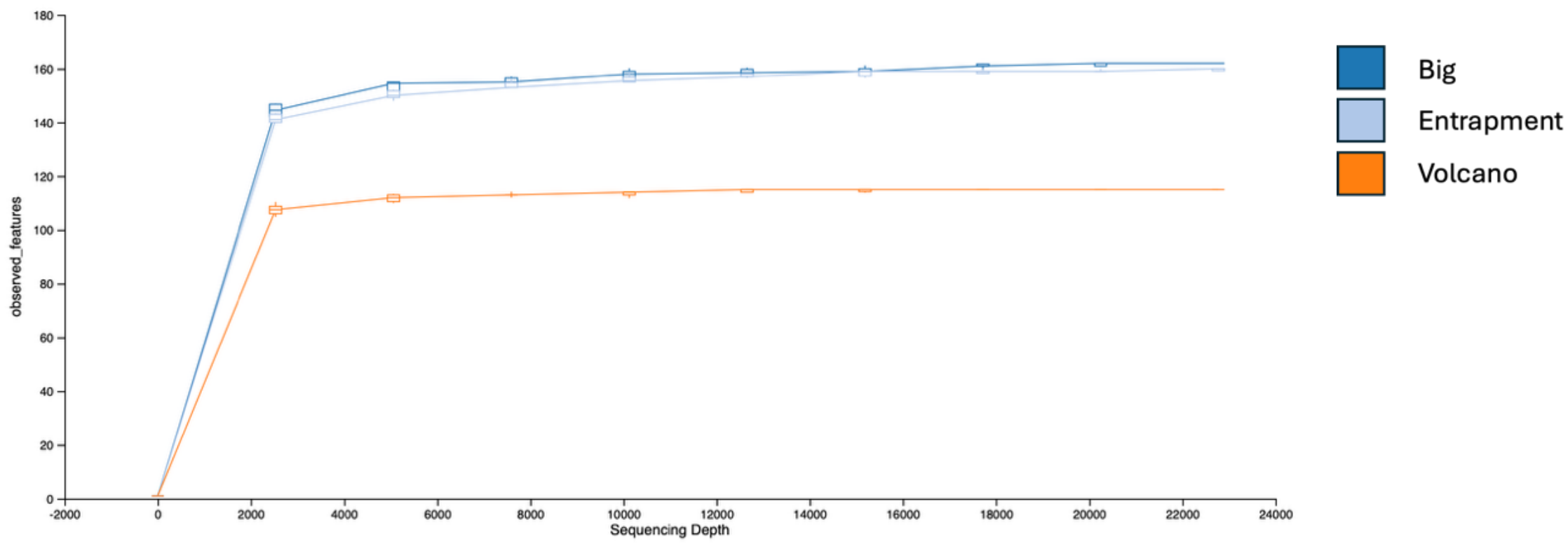

(b)

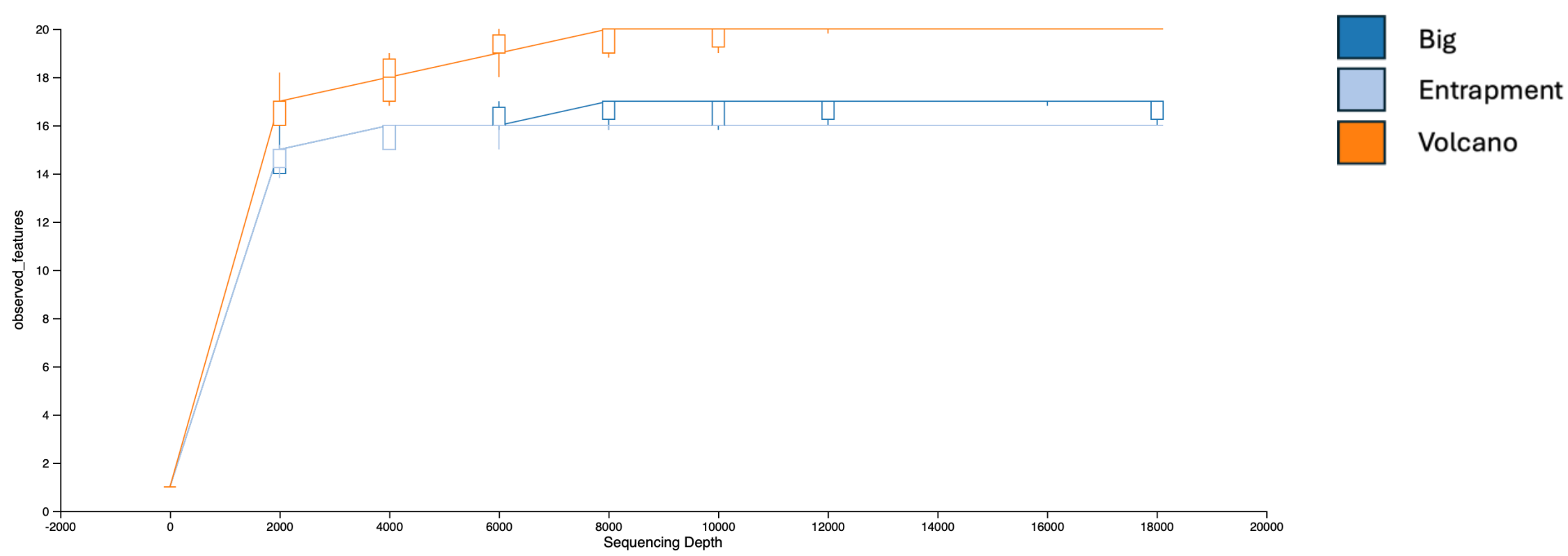

(c)

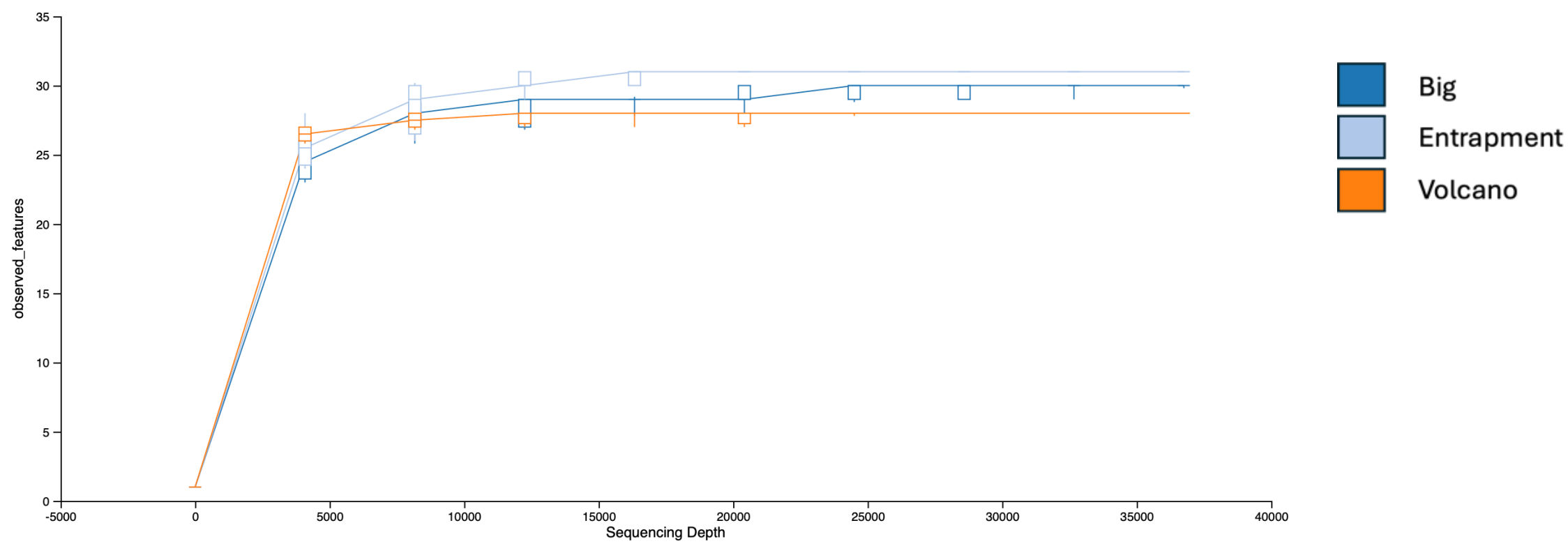

observed_features
Sequencing Depth
35
30
25
20
15
10
5
0
-5000
0
5000
10000
15000
20000
25000
30000
35000
40000
Big
Entrapment
Volcano
