# Supplementary material for: Biosignature reservoirs in the Rozel Point tar seeps at Great Salt Lake, Utah
Source: Front Microbiol. 2026 Aug 3;17:1832944. doi: 10.3389/fmicb.2026.1832944 (PMC13479432; doi:10.3389/fmicb.2026.1832944)
Supplement: Supplementary file 2 [file Data_Sheet_3.pdf]

Martin/Sanchez et al. Biosignature Reservoirs in the Rozel Point Tar Seeps
Supplementary Data 3
PCoA analyses

PCoA analyses of the prokaryotic community composition similarity across sampling locations. Locations are color coded according to the figure legend.

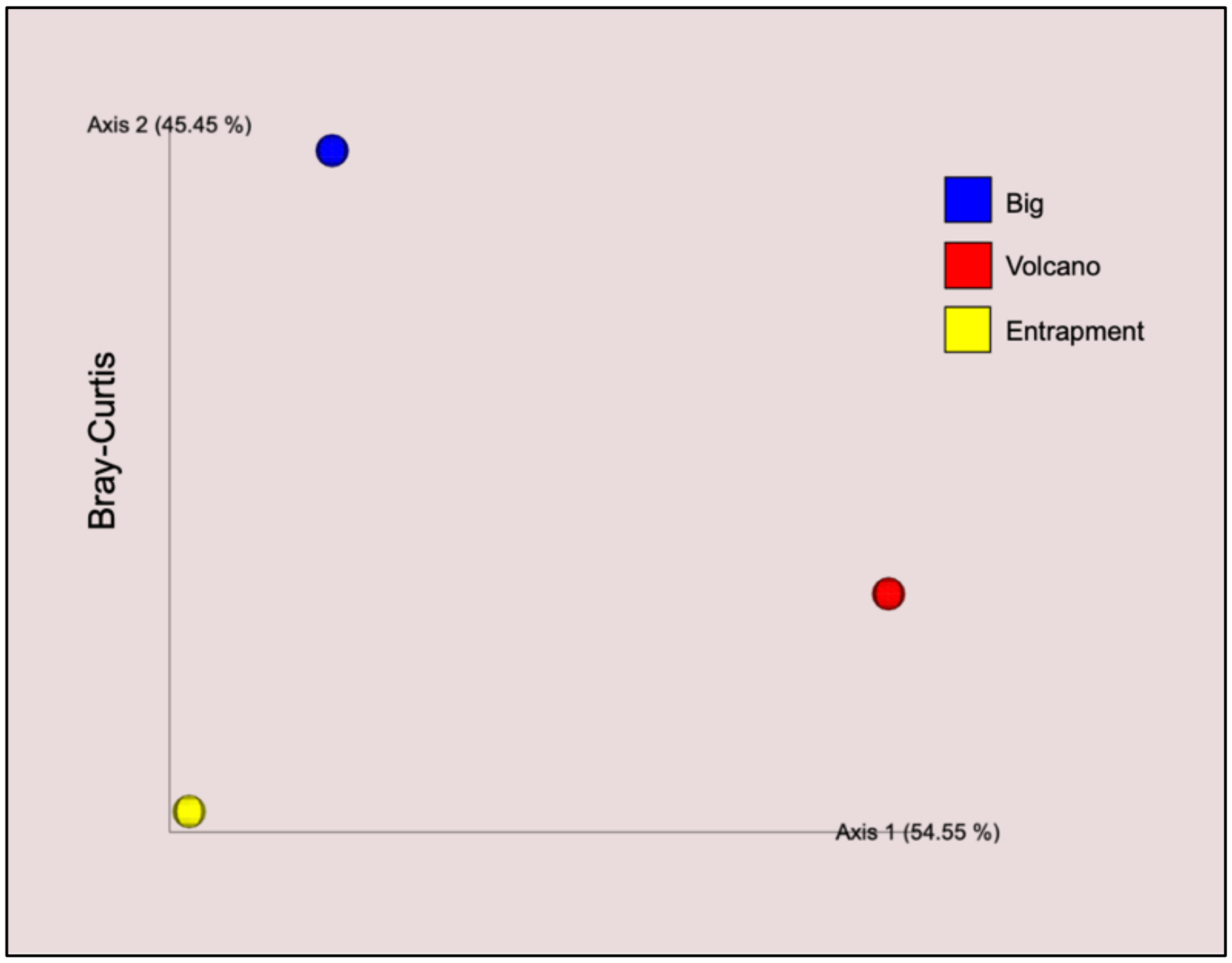

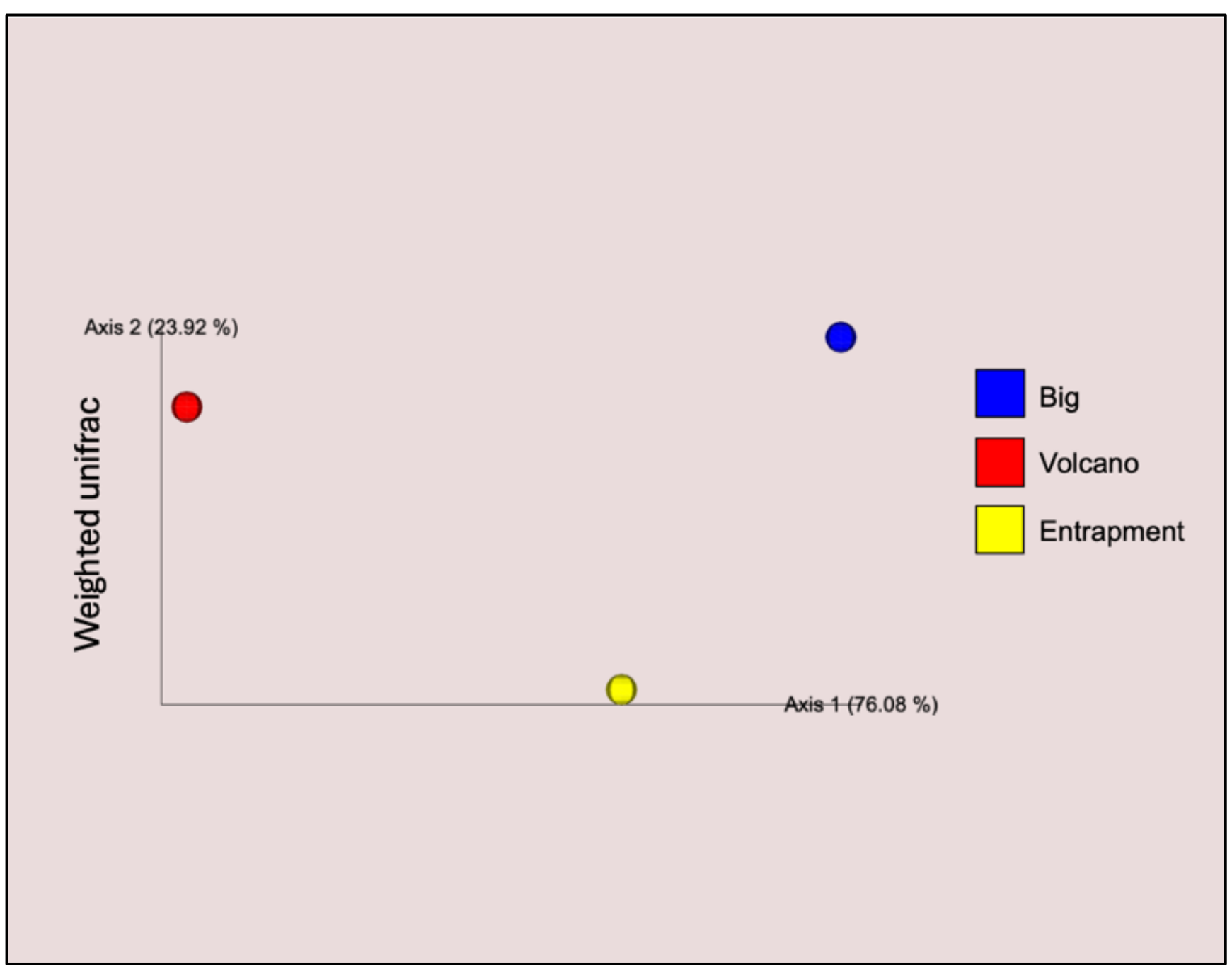

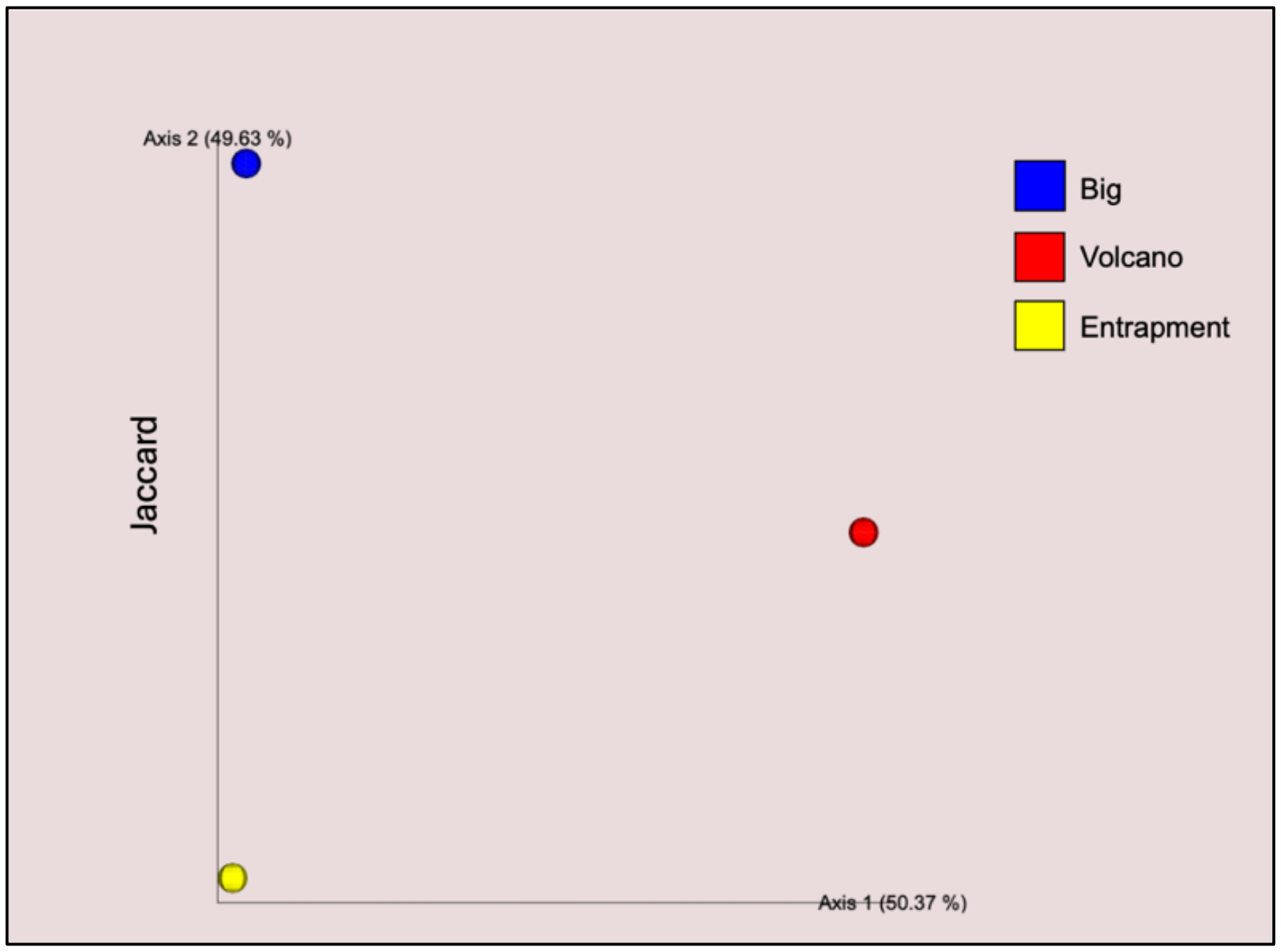
Jaccard
Axis 2 (49.63 %)
Axis 1 (50.37 %)
Big
Volcano
Entrapment

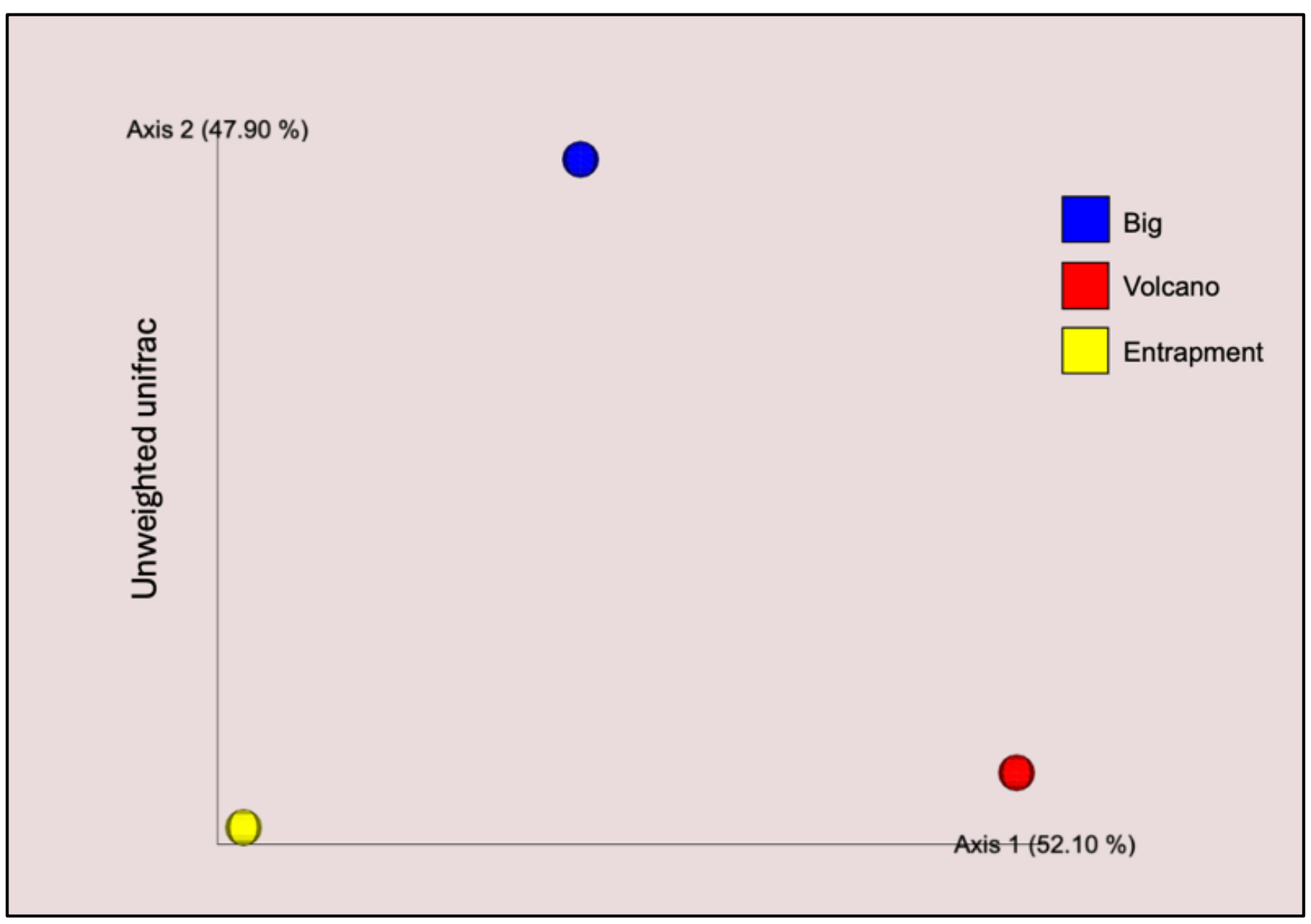
Unweighted unifrac
Axis 2 (47.90 %)
Axis 1 (52.10 %)
Big
Volcano
Entrapment
